# Between care and collapse: the hidden health and emotional struggles of Saudi mothers of children with Down syndrome

**DOI:** 10.3389/fpsyg.2026.1742620

**Published:** 2026-02-13

**Authors:** Waleed Alabri

**Affiliations:** Department of Special Education, College of Education, King Faisal University, Al-Ahsa, Saudi Arabia

**Keywords:** Down syndrome, emotional challenges, health challenges, qualitative research, Saudi Arabia, Saudi mothers

## Abstract

Caring for a child with Down syndrome represents a profoundly complex life experience that demands constant presence and sustained effort from mothers, often becoming a dual source of fulfillment and exhaustion. As daily responsibilities accumulate alongside continuous medical follow-ups, the resulting pressures gradually undermine mothers’ overall balance and well-being. This study seeks to deepen the understanding of this intricate experience by exploring the intertwined health and emotional struggles encountered by mothers while caring for their children with Down syndrome. A qualitative research design was adopted, employing semi-structured interviews with fifteen mothers of children diagnosed with Down syndrome who were enrolled in a rehabilitation center. Data were collected and analyzed thematically to identify core patterns that reflect the enduring pressures associated with long-term caregiving. The analysis revealed two overarching themes: health challenges, which encompassed the sub-themes of persistent physical fatigue and limited preventive care; and emotional challenges, which included continuous emotional strain and fluctuating family support. The findings underscore that the prolonged demands of caregiving impose an escalating burden that significantly affects mothers’ physical balance and emotional well-being, highlighting the need for comprehensive, integrative interventions that strengthen mothers’ ability to sustain their caregiving role effectively and with greater stability.

## 1 Introduction

Down syndrome is recognized as the most prevalent genetic disorder among live births worldwide and remains one of the leading causes of intellectual disability in childhood due to its direct impact on cognitive development, academic achievement, and social interaction in children (Dalrymple et al., 2022). Its influence extends beyond cognitive limitations to affect several vital bodily systems, particularly the nervous and muscular systems, and is frequently accompanied by congenital heart defects that add complexity to diagnosis and clinical management, necessitating close medical follow-up (Mahmood and Gul, 2023). In addition, children with Down syndrome often experience a range of comorbid health conditions, including sleep apnea, epilepsy, and visual or auditory impairments. These health-related pressures require comprehensive care plans grounded in multidisciplinary interventions to ensure quality treatment (Chiracu et al., 2023).

From a psychosocial perspective, Marchal et al. (2017) note that the distinctive physical and facial features of children with Down syndrome tend to attract excessive attention from those around them, which can negatively affect their social integration and sense of acceptance. Similarly, Gashmard et al. (2020) emphasize that this group faces persistent emotional and social challenges that interfere with daily participation and adversely impact psychological well-being. Given their slower developmental trajectory compared to peers, these children typically require sustained therapeutic, educational, and social support, along with expanded opportunities for effective and lasting social inclusion (Soltani and Esbensen, 2024).

Despite individual variations in ability among children with Down syndrome, ongoing developmental delays and associated chronic conditions often limit their independence and reduce their engagement in everyday social activities (Alqahtani et al., 2024). Waugh et al. (2025) add that the persistence and severity of these comorbidities across developmental stages shape lifelong trajectories that intensify caregiving responsibilities within families. In alignment with these findings, Mohammed Elmwafie et al. (2022) assert that delayed developmental progress necessitates continuous therapeutic support and increases dependence on caregivers, imposing additional burdens on the family.

Globally, the literature indicates that the prevalence of Down syndrome is approximately ten cases per 10,000 live births (Shahzad and Manzoor, 2025). In Saudi Arabia, recent statistics estimate one case for nearly 554 live births (Alkahtani, 2022). This rate is influenced by several contextual factors, most notably the religious restrictions surrounding the termination of pregnancies following prenatal genetic diagnosis (Alabri, 2023). Moreover, cultural and social norms that encourage childbirth at advanced maternal ages—particularly during the fourth decade of life—contribute to the higher prevalence of this condition within the Saudi population (Yaman et al., 2025).

Advancements in healthcare services have played a crucial role in reducing infant mortality rates and improving life expectancy among individuals with Down syndrome, enabling many to reach adulthood and experience relative stability (Alabri, 2017). However, this medical progress has also introduced new challenges, particularly the need for long-term care planning within service systems that may lack adequate specialized expertise and sufficient resources (Goldie et al., 2024). Internationally, children with Down syndrome continue to face significant disparities in access to essential services and opportunities for community inclusion. These gaps are further exacerbated by limited public awareness and the absence of sustainable support initiatives (McGlinchey et al., 2025).

## 2 Rationale for the study

A child with Down syndrome grows within a family context that serves as the primary source of support for their health and developmental needs. Within this framework, the mother assumes a central and highly interactive role in the child’s daily care. Although much of the literature emphasizes the medical and rehabilitative aspects of Down syndrome, the direct implications of these demands on maternal physical and emotional well-being remain insufficiently explored, particularly within family-centered societies characterized by high caregiving expectations (AlAhmari et al., 2022).

As the child’s health condition requires ongoing medical monitoring and participation in multiple therapeutic sessions, mothers often find themselves navigating a cycle of repetitive physical strain that gradually affects their rest patterns, sleep quality, and overall energy levels. Opportunities for personal time and self-care become increasingly limited, leaving the body under constant physical and psychological pressure. Concurrently, emotional stressors arise, including persistent fear of deterioration of the child’s health and the absence of reliable social support systems to alleviate daily caregiving burdens (Alqahtani et al., 2024).

Mothers also encounter social challenges shaped by others’ attitudes and responses toward disability, which can negatively influence their sense of acceptance and self-worth. The lack of structured support networks or sustainable community resources amplifies household responsibilities, as caregiving, supervision, and emotional guidance intertwine within the domestic sphere. In conservative cultural contexts, these pressures are magnified by traditional expectations that mothers should provide unwavering care without complaint or respite (Alabri, 2023).

From this standpoint, the present study seeks to provide a deeper understanding of the hidden health and emotional dimensions that characterize the daily experiences of Saudi mothers caring for children with Down syndrome. It aims to highlight how these challenges affect family equilibrium and quality of life while supporting efforts to design more comprehensive and sustainable interventions and to promote policies that strengthen mothers’ resilience and confidence in maintaining long-term caregiving roles.

## 3 Materials and methods

### 3.1 Design

The qualitative approach is considered one of the most suitable methodologies for studies that aim to explore human experiences in depth and interpret individual perceptions within their natural contexts, as it provides flexibility in understanding complex phenomena and constructing meaning grounded in real-life situations (Creswell, 2009).

Guided by this perspective, the present study adopted a qualitative design to explore the health challenges and emotional challenges experienced by Saudi mothers caring for children with Down syndrome. The research was conducted within an interconnected familial and everyday context that reflects the continuous responsibilities mothers assume, allowing for a comprehensive understanding of the caregiving experience (Maxwell, 2013).

Semi-structured interviews served as the sole data collection instrument in this study. This exclusive reliance on interviews ensured methodological coherence and allowed for a direct, in-depth exploration of the mothers’ lived experiences. The flexibility of the semi-structured format enabled participants to articulate their thoughts freely while maintaining alignment with the study’s core objectives, resulting in rich, contextually grounded data suitable for thematic interpretation (Cohen et al., 2011).

### 3.2 Participants

This study utilized purposive sampling to select a group of mothers through a specialized center for special education and rehabilitation in Dammam, located within a culturally diverse environment in the Eastern Province of Saudi Arabia. The selection process focused on recruiting mothers whose experiences aligned closely with the study’s objectives, while ensuring diversity in social backgrounds to capture a broad range of familial experiences and enhance the depth and credibility of the data.

In collaboration with the center’s administration, specific inclusion criteria were established: participating mothers had to be the primary caregivers of a child diagnosed with Down syndrome between the ages of six and sixteen. Participation required prior consent from the mother and formal approval from her spouse, consistent with local cultural norms. Cases that did not meet these criteria were excluded to ensure that the final sample provided rich, relevant, and reliable qualitative data.

The participating mothers ranged in age from 30 to 50 years. Educational backgrounds varied, with seven mothers holding secondary education certificates, one holding a diploma, six holding bachelor’s degrees, and one having completed postgraduate studies at the master’s level. Regarding employment status, eight mothers were employed, while seven were either homemakers or had taken early retirement. Most families had between two and five children, reflecting diverse household structures. The children with Down syndrome represented a broad developmental range, with ages spanning from early childhood to mid-adolescence (6–16 years).

All participating children were enrolled in the same specialized rehabilitation center. The center operates as a private institution supported through a government-funded voucher system, whereby educational and therapeutic fees are covered by official governmental support. In accordance with voucher requirements, the center provides comprehensive support services, including speech therapy, occupational therapy, physical therapy, individualized educational sessions, and structured skills-based programs. Children attended the center on a full-time basis, receiving morning educational and rehabilitation services 5 days per week.

Participant recruitment was facilitated through the center’s supervisor, who initially identified eligible mothers based on the study’s inclusion criteria and obtained their preliminary consent to cooperate with the researcher. Following this step, the researcher contacted each mother individually to provide detailed information about the study and confirm voluntary participation. Mothers were informed that their involvement was entirely optional, that they had the right to withdraw at any stage, and that participation or refusal would not affect the services provided to their children. All interviews were conducted individually at the rehabilitation center, ensuring a familiar and comfortable setting for participants.

The focus on mothers was based on their central role in supervising child care and managing household responsibilities within Islamic societies (Murray and Lawrence, 2000). Although fieldwork involving women in Saudi Arabia can be influenced by complex cultural and social considerations, the researcher’s professional collaboration with the center’s supervisor—along with prior acquaintance with several mothers—facilitated access to participants and strengthened the trust necessary for effective data collection.

A total of fifteen mothers participated in the study, providing authentic and nuanced narratives that captured the realities of family life within the local context. Continued collaboration with the center allowed for the inclusion of additional participants if needed; however, data saturation was achieved with the fifteenth interview, consistent with Guest et al. (2006), who noted that major themes in qualitative research typically emerge within the first six interviews, with full saturation usually reached by approximately the twelfth.

### 3.3 Ethical considerations

The study commenced after obtaining formal ethical approval from the Ethics Committee of King Faisal University (Ref. No: KFU-2025- ETHICS3770), along with official authorization from the Center for Special Education and Rehabilitation. The researcher coordinated in advance with the center’s administration to facilitate procedures and ensure smooth fieldwork implementation. The center’s management retained the mothers’ contact information, which allowed for preliminary communication and introductory meetings to explain the study’s objectives and participation process. All participants provided their informed verbal consent, as written consent forms were deemed culturally inappropriate due to the sensitivity surrounding official documents in the local context and the participants’ preference for maintaining privacy.

Strict confidentiality protocols were observed throughout the study. Each participant was assigned a unique identification code, and no personally identifiable information was recorded. The mothers were informed that all data would be used solely for academic research purposes and that their identities would remain anonymous at all stages of the study. Participation was entirely voluntary, and the introductory meetings served to clarify the study’s aims and potential benefits for families and the broader community. Mothers were encouraged to ask questions at any time, and the researcher responded transparently while reminding them of their right to withdraw from the study at any stage without any consequences or obligations.

### 3.4 Data collection

Face-to-face interviews were conducted systematically with a diverse group of mothers representing varied social, cultural, and educational backgrounds. Their children with Down syndrome also differed in age, gender, health status, developmental needs, and emotional profiles. Semi-structured interviews were selected as the sole data collection method because they provide a carefully balanced structure that combines flexibility with methodological rigor, allowing mothers to express their lived experiences openly and authentically. This approach yielded rich, detailed qualitative data that formed a solid and reliable foundation for understanding mothers’ caregiving experiences within the local sociocultural context (Patton, 2002).

Despite the flexible nature of the interviews, the researcher adhered to a clearly defined methodological framework guided by a set of open-ended questions addressing core themes such as family readiness, social support roles, and their perceived effects on family life. Additional probing questions were used to deepen discussions around issues that were either unanticipated or required further elaboration, thereby broadening the scope and depth of understanding. The shared cultural background between the researcher and the participants fostered a respectful and trusting atmosphere, which enhanced both the authenticity of the narratives and the interpretive accuracy of the data.

Each interview lasted between 25 and 40 minutes. For cultural and personal reasons, audio recording was not used, in alignment with Patton’s (2002) recommendation that avoiding electronic devices can create a more comfortable and open environment for participants. Instead, the researcher meticulously documented the responses by hand, following a structured note-taking procedure. Participants were later given the opportunity to review their transcripts to verify accuracy and clarify any ambiguous points. The researcher also explained that selected excerpts would be translated into English for inclusion in the study report, reinforcing transparency and academic integrity.

### 3.5 Data analysis

The textual data obtained from the mothers’ interviews were transcribed into an electronic document using Microsoft Word, ensuring complete confidentiality by assigning each participant a unique identification code instead of using their real names or any personally identifiable information. The files were stored securely on the researcher’s password-protected device, with an encrypted backup maintained on a trusted cloud storage service to safeguard data integrity and ensure anonymity throughout all stages of analysis.

The researcher employed thematic analysis as the primary analytical framework (Hammersley and Atkinson, 2007). Review of the transcripts began immediately after each interview, allowing for early identification of recurring issues and enabling their further exploration in subsequent sessions. This iterative approach facilitated the development of preliminary patterns that were refined progressively as the data accumulated. Continuous engagement with the texts enhanced analytical depth and supported the formulation of findings grounded in the social and cultural context of Saudi mothers (Ritchie and Spencer, 2002).

As emphasized by Hammersley and Atkinson (2007), thematic analysis is a dynamic process that involves constant movement between the data and emerging meanings. This was implemented through manual coding, whereby the data were systematically categorized under two main themes, each encompassing interconnected sub-themes. This structured organization enabled the identification of similarities and variations across participants’ experiences, while allowing the refinement of preliminary concepts into coherent, well-defined patterns.

To illustrate this analytical process, responses addressing children’s speech difficulties were classified under the first sub-theme, “Persistent Physical Fatigue,” which stemmed from the main theme “Health Challenges.” Table 1 presents the detailed coding framework, outlining the main and sub-themes along with participant identifiers corresponding to each analytical category.

**Table 1:** Data analysis: data coding.

Main themes	Sub-themes	Participants code numbers
Main theme 1: health challenges	Sub-theme 1: persistent physical fatigue	M2, M4, M8, M9, M11, M15
	Sub-theme 2: limited preventive care	M1, M5, M6, M8, M10, M14
Main theme 2: emotional challenges	Sub-theme 1: continuous emotional strain	M2, M5, M7, M10, M12, M13
	Sub-theme 2: fluctuating family support	M1, M3, M4, M7, M9, M15

### 3.6 Qualitative trustworthiness

This study adopted a qualitative framework that employed semi-structured interviews as the sole data collection tool, with a focus on maintaining rigor and trustworthiness across all stages of the research process. The evaluation of quality followed the model of Lincoln and Guba (1985), which is among the most established frameworks for assessing the soundness of qualitative inquiry. This model emphasizes four key criteria: “credibility,” “dependability,” “confirmability,” and “transferability.”

“Credibility” was enhanced through the use of “member checking,” which allowed participants to review their interview transcripts and make necessary clarifications before inclusion in the final analysis. Moreover, the principle of “purposeful diversity” was applied in the sampling process to ensure the inclusion of mothers from varied social, economic, and educational backgrounds, as well as those with children of both genders. This diversity provided a balanced and comprehensive representation of different experiences.

“Dependability” was ensured through the establishment of a systematically documented “audit trail” that detailed each phase of the research process, from the design of the interview guide to data collection and analysis. In addition, “peer debriefing” was conducted in collaboration with the center’s supervisor and consultants from similar institutions to verify the consistency of the interview guide with the study objectives and its suitability to the cultural and social context of Saudi mothers.

“Confirmability” was strengthened by including “direct quotations” from the mothers’ narratives, enhancing transparency and supporting the credibility of the interpretations. The researcher also engaged in “reflexivity,” acknowledging his position as a father of a child with Down syndrome and taking deliberate measures to minimize any personal bias, ensuring that the findings were grounded in authentic participant data.

“Transferability” was achieved by providing a “thick description” of the Saudi context, highlighting the social and cultural dimensions that shape mothers’ lived experiences. Furthermore, the use of a “diverse sample” of mothers and children, differing in age, background, and health conditions, increased the applicability of the study’s findings to similar cultural and regional contexts.

## 4 Results

### 4.1 Theme one: Health challenges

The findings revealed that health-related aspects represent one of the most significant challenges faced by mothers caring for children with Down syndrome. The daily caregiving demands are closely associated with continuous physical exhaustion, accompanied by limited attention to personal health. These pressures often manifest as cumulative fatigue and the absence of regular preventive care, leaving mothers in a state of fragile physical balance that threatens the sustainability of their caregiving effectiveness. From this overarching theme, two sub-themes emerged, each of which will be analyzed in detail in the following sections.

#### 4.1.1 Sub-theme one: persistent physical fatigue

The mothers’ narratives revealed that the caregiving journey extends far beyond the home environment, often involving long and frequent intercity travel to access specialized medical care. This repetitive travel pattern contributes significantly to both physical and mental exhaustion, given the demanding preparations and continuous monitoring of medical appointments. One mother explained: “*It is difficult to explain to our other children that we must travel to another city to see a specialist doctor for my son. The government covers the ticket costs, but we still experience severe physical fatigue on every trip due to the distance, frequency, and constant exhaustion that accompany these journeys*.”

Several mothers described the periods of their child’s illness as the most physically draining, as they intensified their efforts to protect their children from pain. They often spent extended hours providing close supervision and care, which gradually depleted their energy and led to noticeable deterioration in their own physical health over time. As one mother shared: “*I feel extreme exhaustion whenever my daughter becomes ill. I do everything I can to protect her from anything that might worsen her condition. I cannot bear to see her in pain, and that keeps me on constant alert until my body feels like it can no longer endure the fatigue*.”

Sleep disturbance emerged as one of the most common manifestations of physical strain. Mothers reported losing rest due to the need for constant nighttime monitoring, especially during emergencies. The prolonged wakefulness resulted in overall exhaustion that impaired their daily functioning and diminished their caregiving efficiency. One mother described: “*I stayed awake for two consecutive days while my daughter had a high fever. I tried to stay strong, but eventually, I reached a point where I could not keep my eyes open. I told my husband, ‘I am completely exhausted; I need someone to help me for a while*.”

Many mothers added that fragmented sleep had become a normal part of their routine, as they frequently woke up several times at night to attend to their child’s needs. This constant disruption left them in a state of ongoing fatigue, unable to regain full energy or experience genuine rest. In the words of one mother: “*I wake up several times during the night to change my daughter’s clothes or comfort her when she wakes up suddenly. Each time, it is hard to fall back asleep, so I end up sleeping only a few interrupted hours, which leaves me feeling tired throughout the day*.”

#### 4.1.2 Sub-theme two: limited preventive care

Other accounts revealed that chronic anxiety and persistent worry over the child’s health gradually translated into actual physical ailments. Some mothers began to experience chronic conditions directly linked to continuous stress, leaving their physical health increasingly vulnerable to the long-term effects of psychological strain. One mother expressed this experience: “*For some time, I have been suffering from stress caused by my constant effort caring for my daughter, and it has truly affected my health. I developed health problems and take regular medication. What makes it worse is my constant fear that something might happen to me or that my condition will worsen*.”

It was also observed that some mothers neglect their own medical needs even when feeling unwell, believing their family responsibilities prevent them from being absent. As a result, they postpone seeking treatment indefinitely, despite their evident need for care. As highlighted by one mother: “*When I get sick or tired, I do not go to the hospital because I always think, who will take care of my son while I am gone? I have limited help at home. So, I tell myself I will get better on my own, even though I feel weaker day by day*.”

Several mothers explained that their continuous preoccupation with medical appointments for their children often leads them to postpone attending to their own health. They expressed that fear of leaving their child, even for a short period, prevents them from undergoing regular checkups, resulting in the accumulation of untreated health issues and the absence of consistent preventive care. One mother illustrated this challenge: “*I have some chronic health issues, but I only see a doctor when I feel very sick. I always say, after my daughter’s appointments I’ll go, but I never find the time. I feel like I have neglected myself, but I cannot leave her alone even for a single day*.”

Some mothers also noted that the intensity of daily responsibilities made them overlook the importance of self-care, as their focus shifted entirely toward their child’s well-being. Over time, this neglect became a normalized part of their daily routine, often without recognizing its health consequences. One mother reflected: “*I no longer think about myself at all. What matters most is that my son is fine. Even when I feel tired or have a headache, I ignore it and tell myself it will pass. But I have noticed that my body gets tired more easily now, probably because I do not rest or visit the doctor like I used to*.”

### 4.2 Theme two: Emotional challenges

The interviews revealed that emotional burdens represent an equally profound dimension of mothers’ experiences, standing alongside the physical and health-related strains of caregiving. These challenges stem from the mothers’ deep emotional attachment to their children and the fluctuating levels of support and recognition they receive within their families and communities. The emotional strain often manifests as continuous emotional depletion and an inconsistent sense of appreciation for their efforts, amplifying the weight of their daily responsibilities. From this overarching theme, two sub-themes emerged that reflect the broader emotional dimensions of these challenges, each of which will be analyzed separately in the following sections.

#### 4.2.1 Sub-theme one: continuous emotional strain

The mothers explained that daily caregiving responsibilities consume nearly every hour of their day, leaving little room for rest or personal activities. Their accounts illustrated how their routines revolve entirely around their child’s needs, with no time for self-care or balance between family demands and personal well-being. According to one mother: “*It is very difficult to find any free time with my son who has Down syndrome. Almost every aspect of our daily life revolves around him. I never find a moment for myself or even to sit without being occupied. Every minute is dedicated to his care and comfort*.”

Several mothers added that their caregiving roles extend to planning daily activities and constant monitoring, leaving them with almost no time for themselves. They described this pattern as a continuous state of mental and physical preoccupation, in which distinguishing between being a mother and a caregiver becomes nearly impossible. One mother commented: “*There are moments of joy and difficulty in our life with my son who has Down syndrome. Our daily routine centers entirely on his needs. My focus is on meeting his requirements and planning his activities, making him the focal point of our entire day from morning to night*.”

Many mothers shared that their emotional attachment to their children goes beyond what is typical, as any sign of discomfort or illness directly affects their own mental state. This deep bond makes worry and sadness a constant part of their day, creating an ongoing sense of emotional fatigue. One participating mother stated: “*When my daughter cries or gets sick, I feel as if I am the one who is ill. I cannot bear to see her in pain. Even when she sleeps, I stay awake thinking about what might upset her tomorrow. I live in constant anxiety and emotional exhaustion that never ends*.”

Some mothers also described a recurring feeling of psychological depletion after years of continuous caregiving without sufficient rest. The emotional fatigue accumulates silently, eventually turning into an inner sense of emptiness and exhaustion, even as their love and attachment to their children remain strong. One participating mother described: “*Sometimes I smile in front of everyone, but inside I feel indescribably tired. I love my daughter deeply, but I am exhausted. Every day feels the same—routine and worry. Even when she’s fine, I ask myself: how long can I keep enduring this pressure?*”

#### 4.2.2 Sub-theme two: fluctuating family support

Several mothers pointed out that the absence of recreational and sports activities outside rehabilitation centers adds to their psychological burden, as there are few suitable spaces that allow their children to participate and integrate into supportive environments. This lack of inclusion often leaves families feeling isolated and deprived of emotional relief opportunities for both themselves and their children. As one participating mother put it: “*At present, my daughter has no place to go except the rehabilitation center. There are no other sports activities she can join, even though such activities would help her release her energy and ease the pressure on us as a family. Only one cultural center in the region offers such programs*.”

In other cases, mothers expressed frustration about their children being excluded from public activities, particularly sports, due to their limited physical abilities compared to other children. This unintended form of discrimination restricts opportunities for social inclusion and reinforces the child’s sense of being different from peers. One mother explained: “*Participation in activities, especially sports, is very important for my son, but the local park only allows children who are more physically capable to join. They exclude my son because he has Down syndrome, even though he really wants to play with them*.”

Conversely, some mothers observed that enrolling their children in rehabilitation centers brought noticeable improvements to their emotional well-being, providing a supportive educational environment that enhanced their confidence and daily happiness. These experiences highlight the vital role of specialized institutions in improving children’s psychological and social quality of life. As one mother shared: “*My daughter becomes extremely happy when getting ready to go to the center, and as soon as she arrives, she starts hugging everyone. You can see her joy from her smile whenever we mention the center’s name. There is a clear difference between her mood when she used to stay at home and now that she attends regularly*.”

One mother shared her long search for a suitable rehabilitation center, explaining that finding an appropriate educational environment greatly reduced the family’s stress. Despite the long commute, the child’s emotional stability and happiness made the daily effort worthwhile. One mother described: “*We searched for a long time to find a proper rehabilitation center for my daughter. After many attempts, we found this one that meets her needs, even though it’s far from our home. Still, she’s happy there, especially since support is provided by local authorities, which eases a lot of our burden*.”

## 5 Discussion

This section discusses the findings of the study in light of its primary aim: to explore the health and emotional struggles experienced by mothers while caring for children with Down syndrome. The discussion integrates relevant literature to interpret the results and compare their implications with the broader social and cultural contexts that shape maternal caregiving experiences.

### 5.1 Health challenges

Frequent travel to hospitals emerged as one of the primary sources of physical exhaustion among mothers caring for children with Down syndrome, negatively affecting their daily health and stamina. Chiracu et al. (2023) noted that certain forms of physical strain may have a more profound negative impact than emotional stress, making caregiving a complex, multidimensional burden. This finding aligns with Shahzad and Manzoor (2025), who emphasized that caregiving must be examined within the broader framework of physical health, considering variations across age, caregiver characteristics, and the child’s specific needs, as these factors directly influence family stability. Similarly, Carrada et al. (2020) highlighted that spousal and familial support play a crucial role in helping mothers cope with the physical strain associated with raising a child with a disability.

Building on this perspective, the physical toll of caregiving among mothers of children with Down syndrome appears to have direct and cumulative effects on maternal health, with ongoing stress increasing the likelihood of physical deterioration and lower health-related well-being. Lappé et al. (2018) reported that chronic stress accumulation contributes to physical fatigue among caregivers, making them more susceptible to exhaustion. Staunton et al. (2023) further explained that the negative effects of such pressures extend beyond the mothers themselves, affecting the children and the family system as a whole, underscoring the need for preventive and holistic family-centered interventions. In the same context, Gashmard et al. (2020) stressed the importance of establishing specialized support programs that address the physical burdens of caregiving, thereby enhancing mothers’ ability to sustain their caregiving roles while maintaining household stability.

Consequently, the mothers in this study frequently exhibited symptoms of disrupted sleep and persistent fatigue, reflecting a diminished capacity for physical recovery. Marchal et al. (2017) found that mothers of children with Down syndrome typically obtain fewer hours of sleep compared to others, impairing their efficiency in daily caregiving tasks and destabilizing family balance. Esbensen et al. (2021) similarly observed that children’s sleep disturbances are closely linked to parental sleep quality, directly affecting cognitive health and overall well-being. Complementing these findings, Fullwood et al. (2025) reported that caregivers often suffer from inadequate rest, reduced physical activity, and irregular eating patterns, while also delaying their own medical appointments, which ultimately contributes to greater social isolation.

### 5.2 Emotional challenges

The lack of sufficient time for social activities emerged as one of the central emotional burdens, negatively affecting the mothers’ overall quality of daily life and emotional well-being. This finding aligns with Marchal et al. (2013), who observed that families raising children with developmental disabilities experience higher levels of emotional exhaustion, particularly in relation to social functioning and daily vitality. Similarly, Xanthopoulos et al. (2017) highlighted that having a child with Down syndrome often imposes cumulative psychological and social burdens on mothers, restricting their ability to maintain active social networks and disrupting their emotional balance. In addition, Malak et al. (2015) found that lower behavioral and social performance among children often compels mothers to spend extended hours on direct caregiving, further intensifying pressures associated with reduced social participation.

In interpreting these emotional challenges, it is essential to consider the broader cultural and religious context shaping maternal caregiving experiences in Saudi society. Within collectivist cultures, motherhood is closely tied to moral responsibility, family honor, and religious meaning, which may intensify emotional pressure while simultaneously fostering resilience. Previous research has shown that religious beliefs often function as a central coping mechanism among mothers of children with disabilities, promoting acceptance, patience, and psychological endurance (Mohamed Madi et al., 2019). Similarly, qualitative studies conducted in Middle Eastern contexts indicate that spiritual interpretations of disability, such as perceiving caregiving as a form of divine test or reward, significantly influence maternal emotional adjustment (Dababnah and Parish, 2013). However, cultural expectations surrounding maternal responsibility may discourage mothers from seeking emotional support, while prolonged caregiving demands can contribute to cumulative emotional fatigue over time (Mohamed Madi et al., 2019). These cultural dynamics help explain the complex emotional experiences identified in the present study and highlight the importance of culturally responsive psychosocial support for mothers of children with Down syndrome.

In light of these cultural influences, the findings of the present study further reveal that the absence of inclusive community programs for children with Down syndrome outside rehabilitation centers significantly limits participation opportunities and negatively impacts family life as a whole. AlAhmari et al. (2022) similarly emphasized that the lack of accessible activities amplifies mothers’ burdens alongside their other domestic obligations, constraining their capacity to balance caregiving with social involvement, which consequently affects their psychological well-being. Consistent with this, AlShatti et al. (2021) demonstrated that community participation is closely linked to a child’s functional and intellectual abilities, making the scarcity of supportive programs an additional obstacle to meaningful inclusion. Steffensen et al. (2022) further observed that family life in such contexts is marked by a blend of challenges, joy, frustration, and contentment, reflecting the inherent complexity of these experiences and reinforcing the need for supportive initiatives that enhance quality of life.

Accordingly, discussions revealed that children’s enrollment in rehabilitation centers yields multiple benefits for both the child and the family, as it promotes skill development, strengthens social interaction, and provides psychological support. Darla and Bhat (2021) found that when parents adopt positive coping strategies, overall family well-being improves, enhancing both the living conditions and the emotional climate for children and caregivers. Likewise, Nawi et al. (2013) reported that early intervention programs, including prenatal support, not only improve cognitive and adaptive abilities among children with Down syndrome but also foster stronger social support networks and more positive parental attitudes, ultimately benefiting child development and behavior. In parallel, Barbu et al. (2021) affirmed that families’ resilience improves when targeted family-strengthening programs are implemented, emphasizing the critical role of caregiver counseling in promoting engagement and adaptability.

The findings collectively demonstrate that mothers’ caregiving experiences involve interconnected physical and emotional challenges, thereby directly addressing the study’s aim of understanding maternal health and emotional struggles within the context of raising children with Down syndrome.

The findings of this study carry important practical implications for healthcare providers, rehabilitation centers, and family-support services working with families of children with Down syndrome. The identified physical and emotional challenges highlight the importance of adopting integrated support approaches that consider mothers’ health, psychological well-being, and caregiving capacity as interconnected domains. Incorporating maternal-centered perspectives into service planning may contribute to more sustainable caregiving environments and improved family stability.

## 6 Limitations and future research

This study offers significant scholarly value as a qualitative inquiry into the health challenges and emotional challenges experienced by mothers while caring for children with Down syndrome. Grounded in first-hand narratives, it captures the multifaceted dimensions of physical exhaustion and psychological strain embedded in mothers’ daily lives. Nonetheless, the interpretation of its findings should be considered in light of certain limitations, along with recommendations for future research to further advance understanding in this field.

1. The findings of this study have limited generalizability due to the small sample size, as the research prioritized depth of experience over numerical breadth. Future studies are encouraged to recruit larger and more socio-geographically diverse samples to enhance the representativeness and applicability of the results.

2. Data collection was confined to a specific time frame, which may not fully capture evolving changes in healthcare systems or psychological support services. Longitudinal research is therefore recommended to explore how mothers’ health-related and emotional burdens shift over time in response to changing family or service contexts.

3. The study employed a qualitative design based on individual interviews, which naturally reflects subjective perspectives rather than quantifiable outcomes. Subsequent research could integrate quantitative instruments—such as standardized questionnaires—to complement qualitative insights with measurable and comparable data.

4. Finally, future studies should incorporate the perspectives of fathers and other caregivers to provide a more holistic understanding of family dynamics in managing the health and emotional dynamics associated with caring for children with Down syndrome.

## 7 Conclusion and recommendations

The findings of this study reveal a profound interplay between the health challenges and emotional challenges faced by mothers caring for children with Down syndrome. The sustained demands of caregiving extend beyond physical exertion to encompass deep emotional strain, highlighting the fragility of balance between nurturing and exhaustion, between familial duty and personal well-being. These results underscore the urgent need for holistic interventions that promote mothers’ physical and psychological wellness, thereby supporting their sustained ability to provide effective and stable care. In light of these findings, the following key recommendations are proposed.

First, healthcare and therapeutic scheduling systems for children with Down syndrome should be optimized to reduce long waiting periods, thereby alleviating part of the physical and emotional burden borne by mothers during continuous follow-up.

Second, health and psychological support programs specifically designed for mothers should be developed collaboratively between the Ministry of Health and the Ministry of Human Resources and Social Development. Such programs would strengthen mothers’ ability to adapt to the ongoing demands of caregiving and mitigate the cumulative effects of physical fatigue and emotional depletion.

Third, regular community-based initiatives should be implemented in neighborhood centers, including inclusive sports and recreational activities for children with Down syndrome alongside their peers. These programs would help relieve mothers’ psychological stress while fostering emotional balance and social integration.

Finally, recreational institutions and private-sector organizations are encouraged to introduce exemptions and corporate social responsibility initiatives that offer families of children with Down syndrome regular opportunities for leisure participation. Such measures would reduce the psychological load on mothers and enhance their overall well-being and family quality of life.

## Data Availability

The raw data supporting the conclusions of this article will be made available by the authors, without undue reservation.

## References

[ref1] Alabri W. (2017). The inclusion of children with down’s syndrome in mainstream primary schools in Saudi Arabia: understanding the perspective of school principals. [Unpublished Doctoral Dissertation]. Lincoln: University of Lincoln.

[ref2] Alabri W. (2023). Maternal perspectives: the needs of Saudi families of children with down’s syndrome. J. Intellect. Disabil. 27, 221–237. doi: 10.1177/17446295211044406 35144500

[ref3] AlAhmari F. S. Alageel A. F. Aldosari M. A. Bagha M. Y. (2022). The quality of life of parents of children with down syndrome in a tertiary care hospital: a qualitative research study at Saudi Arabia. Ann. Med. Surg. 81:104428. doi: 10.1016/j.amsu.2022.104428, 36147136 PMC9486666

[ref4] Alkahtani F. H. (2022). Down syndrome and its oral effects in Saudi Arabian region: a review of literature. J. Datta Meghe Inst. Med. Sci. Univ. 17, 1013–1018. doi: 10.4103/jdmimsu.jdmimsu_433_22

[ref5] Alqahtani A. S. Algabbani M. F. Alhammad S. A. Alwadeai K. S. Alhusaini A. (2024). Physical activity status and its association with quality of life among children with down syndrome in Saudi Arabia: a comparative cross-sectional study. PLoS One 19:e0297111. doi: 10.1371/journal.pone.0297111, 38346033 PMC10861077

[ref6] AlShatti A. AlKandari D. AlMutairi H. AlEbrahim D. AlMutairi A. AlAnsari D. . (2021). Caregivers’ perceptions and experience of caring for persons with down syndrome in Kuwait: a qualitative study. Int. J. Dev. Disabil. 67, 381–390. doi: 10.1080/20473869.2021.1910780, 34570835 PMC8451671

[ref7] Barbu D. Barbu M. R. C. Brabiescu Călinescu L. Cosma G. Cosma M. A. Forțan C. . (2021). Empowering, communication and motivation for people with intellectual disability in Colpbol sport practice. J. Sport Kinetic Mov. 2:4–13. doi: 10.52846/jskm/38.2021.1.1

[ref8] Carrada C. F. Scalioni F. A. R. Abreu L. G. Ribeiro R. A. Paiva S. M. (2020). Impact of oral conditions of children/adolescents with down syndrome on their families’ quality of life. Spec. Care Dentist. 40, 175–183. doi: 10.1111/scd.12444 31885104

[ref9] Chiracu A. Cosma G. A. Stepan A. R. Cosma M. A. Corlaci I. Călugăru E. D. C. . (2023). Psychological capital, quality of life, and well-being in mother caregivers of individuals with down syndrome. Front. Psychol. 14:1145104. doi: 10.3389/fpsyg.2023.1145104, 36895731 PMC9989283

[ref10] Cohen L. Manion L. Morrison K. (2011). Research methods in education. 7th Edn. New York: Routledge.

[ref11] Creswell J. W. (2009). Research design: qualitative, quantitative, and mixed methods approaches. 3rd Edn. Los Angeles: Sage Publications, Ltd.

[ref12] Dababnah S. Parish S. L. (2013). “At a moment, you could collapse”: raising children with autism in the West Bank. Child Youth Serv. Rev. 35, 1670–1678. doi: 10.1016/j.childyouth.2013.07.007

[ref13] Dalrymple R. A. Somerville L. H. Hamza S. Matta N. (2022). Fifteen-minute consultation: the review of a child with trisomy 21 (down’s syndrome). Arch. Dis. Child. Educ. Pract. Ed. 107, 88–94. doi: 10.1136/archdischild-2020-319814, 33452013

[ref14] Darla S. Bhat D. (2021). Health-related quality of life and coping strategies among families with down syndrome children in South India. Med. J. Armed Forces India 77, 187–193. doi: 10.1016/j.mjafi.2020.07.010, 33867636 PMC8042493

[ref15] Esbensen A. J. Schworer E. K. Hoffman E. K. Wiley S. (2021). Child sleep linked to child and family functioning in children with down syndrome. Brain Sci. 11:1170. doi: 10.3390/brainsci11091170, 34573191 PMC8465298

[ref16] Fullwood K. Collaro A. Power L. Chawla J. (2025). Quality of life and mental health in caregivers of children with down syndrome and sleep problems. J. Appl. Res. Intellect. Disabil. 38:e70103. doi: 10.1111/jar.70103, 40693451 PMC12281471

[ref17] Gashmard R. Ahmadi F. Kermanshahi S. M. K. (2020). Coping strategies adopted by Iranian families of children with down syndrome: a qualitative study. Medicine 99:e20753. doi: 10.1097/md.0000000000020753, 32664068 PMC7360268

[ref18] Goldie M. J. Dobson K. L. Munce S. E. Killackey T. Bayley M. Stapleford C. . (2024). Future care planning of adults with childhood-onset neurodevelopmental disabilities: a scoping review. Res. Dev. Disabil. 154:104843. doi: 10.1016/j.ridd.2024.104843, 39388809

[ref19] Guest G. Bunce A. Johnson L. (2006). How many interviews are enough? An experiment with data saturation and variability. Field Methods 18, 59–82. doi: 10.1177/1525822X05279903

[ref20] Hammersley M. Atkinson P. (2007). Ethnography: principles in practice. 3rd Edn. London: Routledge.

[ref21] Lappé M. Lau L. Dudovitz R. N. Nelson B. B. Karp E. A. Kuo A. A. (2018). The diagnostic odyssey of autism spectrum disorder. Pediatrics 141, S272–S279. doi: 10.1542/peds.2016-4300C, 29610407 PMC5972532

[ref22] Lincoln Y. S. Guba E. G. (1985). Naturalistic inquiry. India: Sage Publications.

[ref23] Mahmood A. Gul A. (2023). Parental stress, familial burden and quality of life in parents of children with down syndrome. J. Prof. Appl. Psychol. 4, 61–70. doi: 10.52053/jpap.v4i1.151

[ref24] Malak R. Kostiukow A. Krawczyk-Wasielewska A. Mojs E. Samborski W. (2015). Delays in motor development in children with down syndrome. Med Sci Monit 21, 1904–1910. doi: 10.12659/MSM.893377, 26132100 PMC4500597

[ref25] Marchal J. P. Maurice-Stam H. Hatzmann J. van Trotsenburg A. P. Grootenhuis M. A. (2013). Health related quality of life in parents of six to eight year old children with down syndrome. Res. Dev. Disabil. 34, 4239–4247. doi: 10.1016/j.ridd.2013.09.011, 24083990

[ref26] Marchal J. P. van Oers H. A. Maurice-Stam H. Grootenhuis M. A. van Trotsenburg A. P. Haverman L. (2017). Distress and everyday problems in Dutch mothers and fathers of young adolescents with down syndrome. Res. Dev. Disabil. 67, 19–27. doi: 10.1016/j.ridd.2017.05.005, 28618319

[ref27] Maxwell J. A. (2013). Qualitative research design: an interactive approach. 3rd Edn. London: Sage Publications, Ltd.

[ref28] McGlinchey E. Fortea J. Vava B. Andrews Y. Ranchod K. Kleinhans A. (2025). Raising awareness and addressing inequities for people with down syndrome in South Africa. Int. J. Equity Health 24:7. doi: 10.1186/s12939-024-02349-3, 39789562 PMC11720340

[ref29] Mohamed Madi S. Mandy A. Aranda K. (2019). The perception of disability among mothers living with a child with cerebral palsy in Saudi Arabia. Glob. Qual. Nurs. Res. 6:2333393619844096. doi: 10.1177/2333393619844096, 31065572 PMC6488775

[ref30] Mohammed Elmwafie S. Ibrahim Abdalla Ibrahim A. Hamdy Abd Elmonem H. Amin Sayed M. Ahmed S. (2022). Effectiveness of coping strategies intervention on quality of life for mothers having children with down syndrome. Egypt. J. Health Care 13, 1696–1714. doi: 10.21608/ejhc.2022.253722

[ref31] Murray L. Lawrence B. (2000). Practitioner-based enquiry: principles for postgraduate research. London: Taylor & Francis Limited.

[ref32] Nawi A. M. Ismail A. Abdullah S. (2013). The impact on family among down syndrome children with early intervention. Iran. J. Public Health 42:996. 26060660 PMC4453896

[ref33] Patton M. Q. (2002). Qualitative research and evaluation methods. 3rd. Edn. London: Sage Publications, Ltd.

[ref34] Ritchie J. Spencer L. (2002). “Qualitative data analysis for applied policy research” in The qualitative researcher’s companion. eds. Huberman A. M. Miles M. B. (London: Sage Publications), 305–329.

[ref35] Shahzad S. Manzoor I. (2025). Quality of life of mothers having children with down syndrome. J. Shalamar Med. Dent. Coll. 6, 22–27. doi: 10.53685/jshmdc.v6i1.289

[ref36] Soltani A. Esbensen A. J. (2024). Role of child demographic, executive functions, and behavioral challenges on feelings about parenting among parents of youth with down syndrome. Res. Dev. Disabil. 148:104717. doi: 10.1016/j.ridd.2024.104717, 38479073 PMC11031302

[ref37] Staunton E. Kehoe C. Sharkey L. (2023). Families under pressure: stress and quality of life in parents of children with an intellectual disability. Ir. J. Psychol. Med. 40, 192–199. doi: 10.1017/ipm.2020.4, 32106892

[ref38] Steffensen E. H. Rosvig L. H. Santoro S. Pedersen L. H. Vogel I. Lou S. (2022). Parenting a child with down syndrome: a qualitative study of everyday practices in Danish families. J. Genet. Couns. 31, 758–770. doi: 10.1002/jgc4.1542, 34939262

[ref39] Waugh K. A. Wilkins H. M. Smith K. P. Ptomey L. T. (2025). Charting the future: current and future directions in translational research for individuals with down syndrome. J. Neurodev. Disord. 17:38. doi: 10.1186/s11689-025-09630-8, 40629284 PMC12235967

[ref40] Xanthopoulos M. S. Walega R. Xiao R. Prasad D. Pipan M. M. Zemel B. S. . (2017). Caregiver-reported quality of life in youth with down syndrome. J. Pediatr. 189, 98–104.e1. doi: 10.1016/j.jpeds.2017.06.073, 28751125 PMC5614822

[ref41] Yaman F. K. Ezveci H. Dogru S. Harmanci M. S. Bahçeci P. Gezginç K. (2025). The impact of advanced maternal age on pregnancy complications and neonatal outcomes. J. Clin. Med. 14:5387. doi: 10.3390/jcm14155387, 40807009 PMC12347501

